# Cautionary optimism: caffeine and Parkinson’s disease risk

**DOI:** 10.1186/s40734-016-0037-8

**Published:** 2016-06-06

**Authors:** Leonard L. Sokol, Michael J. Young, Alberto J. Espay, Ronald B. Postuma

**Affiliations:** Department of Neurology, James J and Joan A. Gardner Center for Parkinson’s disease and Movement Disorders, University of Cincinnati, Cincinnati, OH USA; Harvard Medical School, Boston, MA USA; Department of Neurology, L7-305 Montreal General Hospital, 1650 Cedar Avenue, Montreal, QC H3G1A4 Canada

**Keywords:** Parkinson’s disease, LRRK2, Caffeine, Risk factors

## Abstract

Most Parkinson’s disease (PD) patients present without known family history and without a diagnosed prodromal phase, underscoring the difficulty of employing primary (neuroprevention) and secondary (neuroprotection) preventions. In cases of monogenic forms, however, potential gene-carrying family members of a proband could engage in neuroprevention, such as exercise or diet modifications, to attenuate the risk of, or delay, disease development. However, a historical lack of recognized disease-modifying interventions has limited clinicians’ ability to recommend reliable preventive measures in caring for at-risk populations. We briefly analyze the first retrospective study to examine caffeine consumption and PD risk in a LRRK2 R1628P cohort.

## Letter to the Editor

Caffeine is strongly associated with reduced risk of Parkinson’s disease (PD). Meta-analyses [1, 2] suggest that non-users have a higher PD risk in a notably dose-dependent fashion [2]. The underlying mechanism for this apparent effect is unclear. Potential explanations include a true neuroprotective benefit (supported by some animal models finding benefits of A_2A_ antagonism [3]), a symptomatic effect which delays diagnosis (supported by preliminary evidence of motor benefit of caffeine in small randomized controlled trials [4]), reverse confounding (prodromal parkinsonism reduces caffeine intake via changes in tolerability or reward mechanisms such as has been suggested for smoking [5]), or confounding by another unmeasured factor (e.g., the putative Parkinson personality [6]).

Most recently, a new case–control study [7] examined caffeine consumption and PD risk in a gene-carrier (LRRK2 R1628P) cohort of Chinese patients. LRRK2 R1628P is a low-penetrance variant that has been associated with increased PD risk in mainly Asian populations, with an odds ratio (OR) ranging from 1.20 to 2.83 [8]. The study included 378 PD subjects and 434 healthy controls (PD median age: 66 years; controls: 60). For OR calculations, the authors defined the reference group (PD cases: 257; controls: 369) as those with the LRRK2 wildtype allele who had a reported history of caffeine consumption. Gene-carrier caffeine-abstainers had a 15.4 (95 % CI = 1.94,122.3, *n* = 11) OR of PD; by contrast, gene-carrier caffeine-consumers had a lower increased OR of 3.07 (2.02-4.66, *n* = 33). On the basis of these data, the study authors suggest that caffeine intake may be associated with reduced risk of PD development especially in those who are gene-carriers.

Some caveats should be considered when interpreting these results. First, the sample size was insufficient to provide an estimate of a true effect, as within the R1628P sample, there were too few PD cases (*n* = 28; 18 caffeine-consumers and 10 caffeine-abstainers) and controls (*n* = 16; 15 caffeine-consumers and 1 caffeine-abstainer), yielding an imprecise OR (1.94 to 122.3) that clearly overlapped with the OR for gene-carrier caffeine-consumers. Second, recall bias or other sources of measurement error may have influenced the interpretation of the PD environmental risk factor questionnaire. Third, it is unclear what number of multiple hypotheses were tested in this cohort; if dozens of potential gene-environment interactions were queried, the chances of a spurious result are high. It should be cautioned that many gene-environment findings based upon single cohort studies are not reproduced. Finally, the physiologic mechanism(s) governing the putative impact of caffeine in the PD neurodegenerative cascade remains unclear. Recognizing the excitement that the *prima-facie* favorable findings of this study may generate among patients and clinicians, these key caveats should be considered in the course of crafting an evidence-based and patient-centered approach to neuropreventive counseling and care.

While this study was neither definitive nor generalizable, it suggests the possibility that disease subtypes may have different environmental risk profiles. Of note, LRRK2 in PD is characterized by inconsistent deposition of synuclein, and a corresponding lower prevalence of non-motor manifestation such as REM sleep behavior disorder (RBD) and dementia [9]. A recent case control study of patients with idiopathic RBD found that caffeine use did not protect against future development of PD; this is the only prospective study that has ever failed to find a caffeine effect [10]. If there is an RBD subtype of PD that does not respond to caffeine, then might non-RBD subtypes respond even more?

If such a study is replicated in a larger series—which is critical for ascertainment of such gene-environmental interactions—it may have implications for other well-known LRRK2 variants, such as G2019S, which has a calculated disease penetrance of 26 % by age 80 [11]. Might caffeine have a similar interaction? If so, might such patients benefit from using caffeine? Without better evidence, there remain dichotomous perspectives [12, 13] on how to counsel carriers and their families. Research that examines how environmental behaviors might attenuate PD susceptibility is warranted [11], as is further investigation on how to counsel such a vulnerable cohort [14].

## Statement on ethics approval and consent to participate

Not applicable.

## Statement of consent for publication

Not applicable.

## Availability of data and materials

Not applicable.

## Abbreviations

LRRK2: leucine-rich repeat kinase 2
OR: odds ratio
PD: parkinson’s disease
RBD: REM sleep behavior disorder
